# A phase I trial of the pan-ERBB inhibitor neratinib combined with the MEK inhibitor trametinib in patients with advanced cancer with EGFR mutation/amplification, HER2 mutation/amplification, HER3/4 mutation or KRAS mutation

**DOI:** 10.1007/s00280-023-04545-4

**Published:** 2023-06-14

**Authors:** Sarina A. Piha-Paul, Chieh Tseng, Hai T. Tran, Meng Gao, Daniel D. Karp, Vivek Subbiah, Apostolia Maria Tsimberidou, Jitesh D. Kawedia, Siqing Fu, Shubham Pant, Timothy A. Yap, Van K. Morris, Bryan K. Kee, Mariela Blum Murphy, JoAnn Lim, Funda Meric-Bernstam

**Affiliations:** 1 Department of Investigational Cancer Therapeutics (A Phase I Clinical Trials Program), University of Texas MD Anderson Cancer Center, 1515 Holcombe Blvd, Unit 455, Houston, TX 77030 USA; 2 Department of Thoracic, Head and Neck Medical Oncology, University of Texas MD Anderson Cancer Center, Houston, TX USA; 3 Department of Gastrointestinal Medical Oncology, Division of Cancer Medicine, University of Texas MD Anderson Cancer Center, Houston, TX USA; 4 Pharmacy Clinical Programs, Division of Pharmacy, University of Texas MD Anderson Cancer Center, Houston, TX USA; 5 Department of Breast Surgical Oncology, University of Texas, MD Anderson Cancer Center, Houston, TX USA; 6 The Sheikh Khalifa Bin Zayed Al Nahyan Institute for Personalized Cancer Therapy, University of Texas MD Anderson Cancer Center, Houston, TX USA; 7 Pharmacy Pharmacology Research, Division of Pharmacy, University of Texas MD Anderson Cancer Center, Houston, USA

**Keywords:** Neratinib, Trametinib, ERBB family, EGFR, KRAS

## Abstract

**Purpose:**

Aberrant alterations of ERBB receptor tyrosine kinases lead to tumorigenesis. Single agent therapy targeting EGFR or HER2 has shown clinical successes, but drug resistance often develops due to aberrant or compensatory mechanisms. Herein, we sought to determine the feasibility and safety of neratinib and trametinib in patients with EGFR mutation/amplification, HER2 mutation/amplification, HER3/4 mutation and KRAS mutation.

**Methods:**

Patients with actionable somatic mutations or amplifications in *ERBB* genes or actionable *KRAS* mutations were enrolled to receive neratinib and trametinib in this phase I dose escalation trial. The primary endpoint was determination of the maximum tolerated dose (MTD) and dose-limiting toxicity (DLT). Secondary endpoints included pharmacokinetic analysis and preliminary anti-tumor efficacy.

**Results:**

Twenty patients were enrolled with a median age of 50.5 years and a median of 3 lines of prior therapy. Grade 3 treatment-related toxicities included: diarrhea (25%), vomiting (10%), nausea (5%), fatigue (5%) and malaise (5%). The MTD was dose level (DL) minus 1 (neratinib 160 mg daily with trametinib 1 mg, 5 days on and 2 days off) given 2 DLTs of grade 3 diarrhea in DL1 (neratinib 160 mg daily with trametinib 1 mg daily). The treatment-related toxicities of DL1 included: diarrhea (100%), nausea (55.6%) and rash (55.6%). Pharmacokinetic data showed trametinib clearance was significantly reduced leading to high drug exposures of trametinib. Two patients achieved stable disease (SD) ≥ 4 months.

**Conclusion:**

Neratinib and trametinib combination was toxic and had limited clinical efficacy. This may be due to suboptimal drug dosing given drug–drug interactions.

Trial registration ID: NCT03065387.

**Supplementary Information:**

The online version contains supplementary material available at 10.1007/s00280-023-04545-4.

## Introduction

The epidermal growth factor family of trans-membrane receptors (ERBB family) members are effective mediators of normal cell growth and development [1]. The ERBB family of transmembrane receptor tyrosine kinases (RTKs) is comprised of four closely related receptors: epidermal growth factor receptor (EGFR; HER1), human epidermal growth factor receptor 2 (HER2), HER3 and HER4, which homo- or heterodimerize to sequentially relay signals primarily through the phosphoinositide 3-kinase (PI3K)/AKT/ mammalian target of rapamycin (mTOR) and mitogen-activated protein kinase (MAPK) pathways [2]. Aberrant expression of EGFR, HER2 and HER3, whether induced by gene amplification, gene mutation or protein overexpression, is linked to the development of many epithelial cancers [2–13].

Neratinib, a potent oral, irreversible, pan-ERBB inhibitor targets EGFR, HER2, and HER4 at the intracellular tyrosine kinase domains [14]. Neratinib reduces EGFR and ERBB2 autophosphorylation, and their downstream signaling, and inhibits the growth of EGFR- and HER2-dependent cell lines [14, 15]. Despite compelling clinical benefit from neratinib in patients with the appropriate genetic alterations, patients frequently develop resistance resulting in cancer progression and/or relapse [4, 10].

The MAPK pathway, the prominent downstream effector of ERBB signaling, is comprised of rat sarcoma virus (RAS), rapidly accelerated fibrosarcoma (RAF), mitogen-activated protein kinase kinase (MEK) and extracellular signal-regulated kinase (ERK). This pathway is involved in proliferation, survival and differentiation and as a result, deregulation of this pathway contributes to cancer [4, 16, 17]. Preclinical studies have shown that in HER2-overexpressing cells, combining MEK inhibition with neratinib reduced phosphorylated ERK more than either single agent [18]. Further, combination therapy suppressed tumor growth and reduced expression of the Forkhead box transcription factor M1 (FOXM1) in HER2-overexpressing breast cancers resistant to trastuzumab and lapatinib, and suppressed tumor growth and increased progression free survival in patient-derived xenografts of breast, colorectal and esophageal cancers with *HER2* mutations [18, 19].

Additionally, the mutant RAS pathway amplifies the ERBB kinase activity both in vitro and in vivo [20]. Broad inhibition of kinases of the ERBB family by neratinib has been shown to suppress *KRAS* G12D mutant-driven lung tumors and enhance the potency of MEK inhibition by trametinib in a cre-inducible immunocompetent mouse model of autochthonous lung cancer [20]. Similarly, another pan-ERBB inhibitor, afatinib, was shown to reduce the progression of *KRAS* G12D driven lung cancer in preclinical mouse models [21]. Both studies have shown that cancers with KRAS mutations rely on resistance mechanisms that involve signaling through the ERBB network. These mechanisms can be effectively targeted either using a combination of neratinib and trametinib or using a single agent afatinib, irrespective of the expression or mutation of EGFR/ERBB [20, 21]. Herein, we report the feasibility and safety results of a single-center study of neratinib and trametinib in patients with *EGFR* mutation/amplification, *HER2* mutation/amplification, *HER3/4* mutation and *KRAS* mutation.

## Methods

### Study design and dosing

This study was part of an ongoing open-label, non-randomized, single-center, phase I dose-escalation trial, conducted in patients with advanced or metastatic cancer with *EGFR* mutation/amplification, *HER-2* mutation/amplification, *HER-3/4* mutation or *KRAS* mutation (NCT03065387). Neratinib was provided by Puma Biotechnology and trametinib was commercially obtained. Neratinib was orally administered in a continuous 28-day cycle and trametinib was given orally once daily or followed a 5 day on and 2 days off schedule. Compliance was determined through review of pill diaries and unused drugs returned at the end of every cycle. The study was sponsored by Puma Biotechnology and was approved by the Institutional Review Board (IRB) in accordance with the Declaration of Helsinki, Good Clinical Practice, and all federal, state and local regulatory guidelines. Consent was obtained from all patients prior to study enrollment.

The protocol followed a standard 3 + 3 design [22]. Adverse events (AE) were graded based on the National Cancer Institute (NCI) Common Terminology Criteria for Adverse Events, version 4.0 (CTCAEv4.0). Dose limiting toxicity (DLT) was defined as any treatment-related grade ≥ 3 non-hematologic toxicities (except for: grade 3 nausea and vomiting lasting < 72 h with adequate anti-emetic and supportive care; grade 3 diarrhea lasting < 48 h with optimal medical therapy; alopecia; and, electrolyte imbalances that resolved with supportive care); any grade 4 hematologic toxicity lasting more than a week; grade 3 thrombocytopenia with bleeding; neutropenic fever; all other grade 3 non-hematologic toxicity and any study drugs related severe or life-threatening conditions not defined in the CTCAEv4.0. DLT evaluable patients were defined as patient that had received at least 75% of the study drugs in the first cycle (28 days). A patient who discontinued therapy without completing the first tumor assessment (radiographic evaluation approximately 8 weeks after baseline) would be in-evaluable for response assessment. The maximum tolerated dose (MTD) was defined as the highest dose at which no more than 1 of 6 evaluable patients had a DLT.

### Safety assessments

All patients were evaluated for new or worsening adverse events by investigator or qualified designee. The assessments were conducted weekly during the first cycle (28 days), and then monthly from cycle 2 onwards. Evaluation could be conducted at higher frequency if required by the patient’s clinical condition. The assessment included a complete physical examination, eastern cooperative oncology group (ECOG) assessment, and the recording of vital signs. All toxicities were carefully evaluated in terms of grading, seriousness, action taken regarding trial agents and causality to each study agent.

### Study oversight

The study was conducted in compliance with the Declaration of Helsinki, Good Clinical Practice, and all relevant federal, state and local regulatory guidelines and was approved by the MD Anderson cancer center Institutional Review Board (IRB). To ensure adherence to the study procedures and patient safety, the study was monitored by Investigational New Drug (IND) office at MD Anderson Cancer Center. All treatment-related toxicities experienced by the patients, including any cases of early termination, were reported to and reviewed by the IND office before approval of enrollment to subsequent cohorts.

### Eligibility criteria

Key inclusion criteria included patients with advanced solid tumors (not hematologic malignancy), either relapsed after standard therapy or without standard therapy available. Patients must have had one of the following pre-identified somatic molecular aberrations predicted to be activating or pathogenic as performed in the Clinical Laboratory Improvement Amendments (CLIA) environment and suitable for enrollment: *EGFR* mutation/amplification, *HER-2* mutation/amplification, *HER-3/4* mutation or *KRAS* mutation; age 18 years or older; measurable disease by Response Evaluation Criteria in Solid Tumors (RECIST) v1.1; ECOG status ≤ 1; with adequate organ functions including absolute neutrophils > 1500 cells/uL, platelets ≥ 100,000/uL, hemoglobin ≥ 9 g/dL, total bilirubin ≤ 1.5 × upper limit of normal (ULN), serum creatinine < 1.5 × ULN and alanine transaminase (ALT) ≤ 2.5 × ULN (≤ 5 × ULN if with liver metastases); patients that are of child-bearing potential must agree to use adequate contraception. Key exclusion criteria included concurrent chemotherapy treatment; uncontrolled illness such as active infection requiring intravenous (IV) antibiotics; any clinically significant heart conditions or gastrointestinal abnormalities that may alter absorption or inability to swallow pills; skin rash > grade 1; albumin < 3 gm/dL; or, history of retinal disorder, dry eye syndrome or blurry vision not evaluated and cleared by ophthalmology prior to starting treatment.

### Genomic eligibility

All genomic alterations in eligible genes were reviewed by the MD Anderson Precision Oncology Decision Support (PODS) prior to patient enrollment. Alterations were researched within the published literature for any known effect on function, stability, expression, or therapeutic sensitivity. Alterations were then classified for their functional significance and variant-level actionability, as previously described [23].

### Assessment of tumor response

Baseline radiographic imaging (e.g., computed tomography (CT) scan or magnetic resonance imaging (MRI)) was performed within four weeks of the start of treatment. Tumor measurements were performed on patients at baseline and at the end of every two cycles (three cycles after 24 weeks). Measurable target lesions were evaluated for response using RECIST v1.1. For purposes of this report, prolonged stable disease (SD) was defined as lasting ≥ 16 weeks.

### Pharmacokinetics

Pharmacokinetic (PK) analysis for trametinib and neratinib was performed on cycle 1, day 15 with blood samples for neratinib and trametinib collected at pre-dose, 1, 3, 4, 6, 8- and 24-h post-dose. Trametinib and neratinib plasma concentrations were quantified using a validated tandem LC/MS assay as described [24, 25]. PK parameters for trametinib and neratinib were determined using noncompartmental analysis methods using Phoenix WinNonlin 8.1 (Certara USA Inc, Princeton, NJ, USA).

## Results

### Patient characteristics

Between the period of November 2017 and March 2020, a total of 20 patients with advanced solid tumor were enrolled to receive combination therapy with neratinib and trametinib. The demographic and clinical characteristics of the patients are summarized in Table 1. The median age of patients was 50.5 years (range 26–71 years) with the majority being female (60%). The median number of prior systemic therapies was 3 (range 1–11). Five (25%) patients had received prior therapy with either EGFR (*n* = 1) or HER-2 targeted therapies (*n* = 4). The most common malignancies were colorectal cancer (*n* = 10, 50%), ovarian cancer (*n* = 3, 15%) and esophageal cancer (*n* = 2, 10%).

**Table 1:** Baseline demographics and clinical characteristics

Characteristic	NER 160 mg + TRA 1 mg Daily	NER 160 mg + TRA 1 mg 5D on/2D off	All
	*N* = 9	*N* = 11	*N* = 20
Gender, *n* (%)			
Male	3 (33.3)	5 (45.5)	8 (40.0)
Female	6 (66.7)	6 (54.5)	12 (60.0)
Median age (years)	51.0 (26–71)	50.0 (31–70)	50.5 (26–71)
Tumor type, *n* (%)			
Cervical	0	1 (9.1)	1 (5.0)
Duodenal	0	1 (9.1)	1 (5.0)
Non-small cell lung	0	1 (9.1)	1 (5.0)
Ovarian	2 (22.2)	1 (9.1)	3 (15.0)
Colorectal	3 (33.3)	7 (63.6)	10 (50.0)
Esophageal	2 (22.2)	0	2 (10.0)
Salivary gland	1 (11.1)	0	1 (5.0)
Breast	1 (11.1)	0	1 (5.0)
Pan-ERBB alterations, *n* (%)			
EGFR amplification (NGS)	1 (11.1)	1^ (9.1)	2 (10.0)
EGFR overexpression (IHC)	1 (11.1)	0	1 (5.0)
EGFR mutation	1^ (11.1)	2 (18.2)	3 (15.0)
ERBB2 amplification			
By NGS By IHC By FISH	2^ (22.2) 0 3 (33.3)	0 0 2 (18.2)	2 (10.0) 0 5 (25.0)
*ERBB2* mutation	2^ (22.2)	2 (18.2)	4 (20.0)
*ERBB3* mutation	1 (11.1)	0	1 (5.0)
Co-occurring *ERBB* alterations**^**	2 (22.2)	1 (9.1)	3 (15.0)
None	0	5 (45.5)	5 (25.0)
*KRAS* mutation, *n* (%)	5 (55.6)	9 (81.8)	14 (70.0)
Number of prior systemic therapies			
Median	5 (2–11)	3 (1–4)	3 (1–11)
Patients with prior EGFR/HER2 targeted therapy*			
	4	1	5

*N *number, *D* day, *Ner* neratinib, *TRA* trametinib, *EGFR* epidermal growth factor receptor, *ERBB* epidermal growth factor, *IHC* immunohistochemistry, *FISH* fluorescence in situ hybridization, *NGS* next generation sequencing

^*^EGFR targeted therapy includes cetuximab; HER2 targeted therapies include trastuzumab, trastuzumab emtansine, pertuzumab and two investigational agents (a HER-2/4-1BB bispecific and a HER-2 tyrosine kinase inhibitor)

**^**co-occurring alterations include the following: *EGFR* G724S and *ERBB2* Y590C (*n* = 1); HER2 amplification by NGS and *ERBB2* D769Y (*n* = 1); *EGFR* amplification by NGS and *EGFR*_L861R (*n* = 1)

Table 1 summarizes the pre-identified somatic molecular alterations that were deemed activating or pathogenic and enrolled in the study**,** including *KRAS* mutations (*n* = 14; 70%) and pan-*ERBB* alterations (*n* = 15, 75%). *ERBB2* amplification (*n* = 7, 35%) was the most common alteration in enrolled patients with pan-*ERBB* alterations followed by *ERBB2* mutation (*n* = 4, 20%). Co-occurring alterations were reported in three patients including an ovarian patient with somatic *EGFR* G724S and *ERBB2* Y590C mutation; a colorectal patient with *HER2* amplification and *ERBB2* D769Y mutation; and a colorectal patient with co-existing *EGFR* amplification by next generation sequencing (NGS) and *EGFR* L861R mutation. Among the *ERBB2* amplified patients, 5 of seven patients had ERBB2 amplification by fluorescence in situ hybridization (FISH) and 2 patients had amplification confirmed by next generation sequencing (NGS).

### Toxicity assessment and adverse event

Twenty patients received study drugs and were evaluable for safety. A total of 9 patients were treated on dose level 1, with continuous oral administration of 160 mg neratinib in combination with 1 mg of trametinib daily. Three of 9 patients treated on dose level 1 discontinued treatment prematurely and failed to complete 75% of study drug due to clinical progression (*n* = 1), consent withdrawal after 7 doses (*n* = 1) and hospitalization due to anemia unrelated to study drugs (*n* = 1). Six of 9 patients completed at least 75% of study drug during first cycle and were evaluable for DLT. Grade 3 diarrhea were reported as DLT in 2 patients at dose level 1. Therefore, dose level minus 1 (160 mg neratinib daily with 1 mg trametinib 5 days on and 2 days off) was selected as maximum tolerated dose (MTD) per protocol guidance. Eleven patients were treated at dose level minus 1 with 8 patients completing at least 75% of the study drug during the first cycle. Three patients failed to complete 75% of study drug in cycle one due to hospital admission for disease-related conditions (*n* = 2) and withdrawal of consent to seek other treatment options (*n* = 1).

Safety assessments are summarized in Table 2. Treatment-related adverse events (TRAEs) were observed in 95% of patients on study. Seven patients (35%) experienced at least 1 or more grade 3 adverse events that were attributed to study drugs. No patients experienced grade 4 or higher TRAEs. There was a higher frequency and grading of TRAEs reported for neratinib as compared to trametinib. Nineteen of 20 patients (95%) had TRAEs that were attributed to neratinib and 80% of patients had TRAEs that were attributed to trametinib. After the completion of cycle 1, three patients discontinued study treatment in subsequent cycles secondary to toxicity. This includes 2 patients coming off trial due to diarrhea attributed to both study drugs during cycles 4 and 7, respectively, and the third patient withdrew consent due to nausea and vomiting attributed to both study drugs during cycle 2.

**Table 2:** Neratinib and trametinib safety assessment

Safety assessment	NER 160 mg + TRA 1 mg Daily	NER 160 mg + TRA 1 mg 5D on/2D off	All
	*N* = 9	*N* = 11	*N* = 20
DLT, *n* (%)			
	2 (22.2)	0	2 (10.0)
TRAE, *n* (%)			
≥ 1 TRAE	9 (100.0)	10 (90.9)	19 (95.0)
≥ 1 TRAE grade ≥ 3	5 (55.6)	2 (18.2)	7 (35.0)
≥ 1 TRAE related to neratinib	9 (100.0)	10 (90.9)	19 (95.0)
≥ 1 TRAE related to neratinib grade ≥ 3	5 (55.6)	2 (18.2)	7 (35.0)
≥ 1 TRAE related to trametinib	8 (88.9)	8 (72.7)	16 (80.0)
≥ 1 TRAE related to trametinib grade ≥ 3	3 (33.3)	1 (9.1)	4 (20.0)
≥ 1 SAE unrelated to study drugs	5	3	8
≥ 1 SAE related to neratinib	7	1	8
≥ 1 SAE related to trametinib	0	1	1
Treatment discontinuation, n (%)			
≥ 1 TRAE resulting in discontinuation	2 (22.2)	1 (9.1)	3 (15.0)

*N* number, D, day; *Ner* neratinib, *TRA* trametinib, *DLT* dose-limiting toxicity, *TRAE* treatment-related adverse event, *SAE* serious adverse event

The TRAEs of neratinib and trametinib are shown in Table 3. Diarrhea (*n* = 19, 95%) was the most common TRAE followed by rash (*n* = 13, 65%), nausea (*n* = 10, 50%), vomiting (*n* = 6, 30%), fatigue (*n* = 4, 20%), mucositis (*n* = 4, 20%) and anorexia (*n* = 3, 15%). Notably, diarrhea also accounted for the most frequent grade 3 AE, observed in 5 patients (25%). Six serious adverse event (SAE) reports of diarrhea, 1 event of vomiting and 1 event of nausea, that were at least possibly related to study drugs, were reported on study (Supp. Table 1).

**Table 3:** Treatment-related adverse events (TRAEs) of neratinib and trametinib

Adverse event Number (%) *	NER 160 mg + TRA 1 mg Daily *N* = 9		NER 160 mg + TRA 1 mg 5D on/2D off *N* = 11		All *N* = 20	
	G < 3	G3	G < 3	G3	G < 3	G3
Diarrhea	5 (55.6)	4 (44.4)	9 (81.8)	1 (9.1)	14 (70.0)	5 (25.0)
Nausea	5 (55.6)	0	4 (36.3)	1 (9.1)	9 (45.0)	1 (5.0)
Vomiting	3 (33.3)	1 (11.1)	1 (9.1)	1 (9.1)	4 (20.0)	2 (10.0)
Fatigue	2 (22.2)	1 (11.1)	1 (9.1)	0	3 (15.0)	1 (5.0)
Anorexia	1 (11.1)	0	2 (18.2)	0	3 (15.0)	0
Leukocytopenia	1 (11.1)	0	0	0	1 (5.0)	0
Abdominal pain	1 (11.1)	0	0	0	1 (5.0)	0
Thrombocytopenia	1 (11.1)	0	0	0	1 (5.0)	0
Rash	5 (55.6)	0	8 (72.7)	0	13 (65.0)	0
Mucositis	2 (22.2)	0	2 (18.2)	0	4 (20.0)	0
Malaise	0	1 (11.1)	0	0	0	1 (5.0)
Short of breath	0	0	1 (9.1)	0	1 (5.0)	0
Hypertension	0	0	1 (9.1)	0	1 (5.0)	0

*G* grade, *N* number, D day, *Ner* neratinib, *TRA* trametinib

The numbers represent the highest grades assigned. Adverse events deemed at least possibly, probably or definitely related to treatment were graded based on the Common Terminology Criteria for Adverse Events, Version 4 (CTCAE 4.0)

To evaluate whether combination therapy of neratinib with trametinib increased toxicity, we compared the TRAEs of all grades (frequency > 10%) between the monotherapies of neratinib or trametinib reported in previous studies, and the TRAEs observed in our combination therapy study. The single agent neratinib taken at 180 mg, the most comparable dose level, has similar toxicity profile and frequency as combination therapy [27]. Trametinib monotherapy (2 mg) has overlapping TRAEs with combination therapy, albeit at a lower frequency. Supp. Table 3 shows the frequency of grade 3 TRAEs for monotherapies of trametinib or neratinib, and for combination therapy. Combination therapy had increased toxicity with increased instances of grade 3 AEs of diarrhea, nausea and vomiting compared to either single agent neratinib or trametinib.

### Pharmacokinetic (PK) analysis

PK parameters were assessed for neratinib and trametinib from available samples obtained from 8 patients that were dosed at dose level minus 1 (160 mg neratinib daily with 1 mg trametinib 5 days on and 2 days off). The PK parameters are summarized in Table 4 and plasma concentration profiles in Fig. 2. After oral administration, trametinib concentration peaked at 5.6 h with C_max_ of 38.1 ± 12.3 ng/mL and trough concentration (C_24_) was 27.8 ± 12.7 ng/ml. The mean area under the concentration–time curve (AUC) was 732 ± 339 ng*h/mL, mean half-life was 52 ± 38 h, and the oral clearance was 1.55 ± 0.63 L/h. For neratinib, the mean AUC, half-life and oral clearance were 1491 ± 1189 ng·h/mL, 18 ± 6.15 h and 76.7 ± 16.9 L/h, respectively. The mean time to maximum concentration was about 6 h post dose administration with Cmax concentration of 102 ± 79.99 ng/mL. Neratinib trough concentration (C_24_) was 43.99 ± 33.19 ng/mL.

**Table 4:** Neratinib and trametinib pharmacokinetic parameters

PK Variables	Neratinib*	Trametinib*	Trametinib (Infante et al.)^32^		
Dose	160 mg	1 mg	2 mg	2.5 mg	3 mg
Number of patients	8	8	13	16	16
Half-life (h)	18 (9.1–26.8)	52 (12.3–597)	57.6 (4·01–210)	90.2 (58.4–183)	86.1 (15.1–291)
Tmax (h)	5.8 (3–8)	5.6 (2.9–8.3)	1.75 (1–3)	2.0 (1–10.2)	2.05 (0.5–10.0)
Cmax (ng/mL)	102 (9–230)	38.1 (18.4–52.3)	22.2 (14–32·9)	24.1 (12.4–63.2)	33.4 (15.6–60.9)
AUC_0-24_ (ng*h/mL)	1491 (130–3121)	732 (197–1054)	370 (256–500)	410 (215–865)	540 (261–968)
AUC_0-∞_ (ng*h/mL)	3191 (231–5569)	2827 (555–22,297)	n/a	n/a	n/a
Ctau or C_24_ ng/mL	43.99 (3.4–93)	27.8 (8.5–42.2)	12.1 (8.26–16.9)	15.1 (6.86–40.5)	17.9 (7.77–35.5)
Oral CLss (L/h)	76.7 (56.5–93.5)	1.55 (0.95–2.4)	n/a	n/a	n/a
AR	1.67 (1.19–2.16)	13.9 (1.3–14.9)	5.97 (4.03–11.5)	4.19 (1.02–13.1)	5.93 (1.50–18.0)

*n/a* data not available, *h* hours, ng nanograms, *L* liter, n number, *mL* milliliter, *Cmax* maximum plasma concentration, *Tmax* time to achieve Cmax, *AUC*_*0-24*,_ area under the curve time 0 to 24 h; AUC0_-∞,_ area under the curve time 0 to infinity, *Ctau* concentration at the end dosing interval, *CLss* oral clearance at steady state, *AR* accumulation ratio

^*^PK parameters were assessed on patients that were dosed at dose level minus 1 (160 mg neratinib daily with 1 mg trametinib 5 days on and 2 days off). All values are calculated as mean (range)

**Fig. 1:**
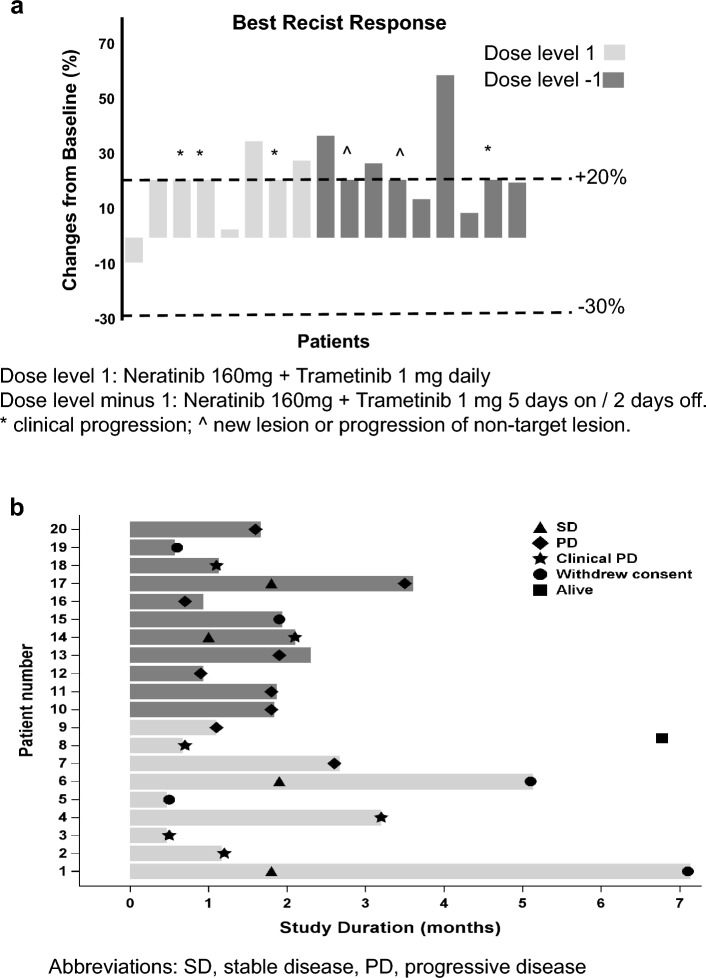
Waterfall plot and swimmer plot depicting response by patient **a** Patients/cohorts are represented by vertical bars on the X-axis. The best RECIST response (%) is depicted on the Y-axis. Seventeen patients were measurable by RECIST 1.1. Four of 17 patients were assigned a value of + 21% for clinical progression (*) and 2 were assigned a value of + 21% for new lesion or PD in non-target lesions (^). The upper dotted line indicates progressive disease by RECIST 1.1 (+ 20% from baseline). The lower dotted line indicates partial response RECIST 1.1 (-30% from baseline). **b** Swimmer plot analysis of 20 patients treated on study. Each lane presents a patient. X-axis represent time from treatment start (months). Y-axis represents patient number. SD, stable disease, PD, progression disease

**Fig. 2:**
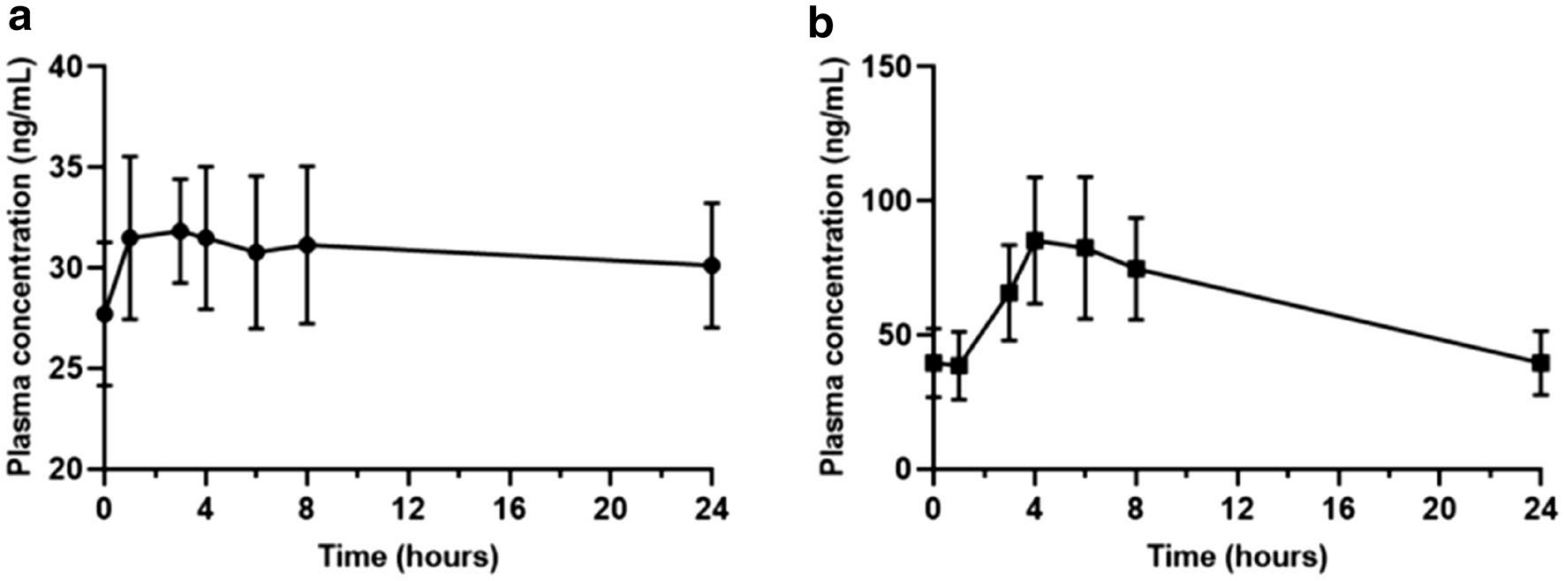
Plasma concentration profiles of (**a**) trametinib and (**b**) neratinib. Mean (standard of deviation) plasma concentrations over time on cycle 1, day 15 (*n* = 8) after co-administration of trametinib 1 mg on 5 days on, 2 days off schedule and neratinib 160 mg daily

### Antitumor activity

Among the 20 treated patients, 17 (85%) were evaluable for efficacy due to having RECISTv1.1 measurable disease and having completed post-baseline tumor assessment by radiographic imaging studies or physician-determined clinical progression. Two patients withdrew from the study during the first cycle to seek other treatment options. One patient was deemed non-evaluable for follow-up tumor re-assessment due to lack of contrast in target lesions to accurately assess the response.

Figure 1a is a waterfall plot depicting best response of these 17 patients. SD ≥ 4 months was observed in 2 patients treated at dose level 1 for a clinical benefit rate of 10% (2/20 patients). These patients included an ovarian cancer patient with *KRAS* G12D and *EGFR* G724S mutations with SD (- 9%) for 7 months and a salivary gland cancer patient with EGFR amplification by IHC with SD (+ 3%) for 5 months. Both patients eventually withdrew consent from study due to treatment-related diarrhea. Figure 1b shows the treatment duration of patients by swimmer plot. The median treatment duration for all patients was 1.8 months (95% CI 1.69 to 2.40 months). Four patients had stable disease by RECIST1.1, however, 2 patients failed to reach prolonged stable disease which we defined as greater than or equal to 4 months duration. As of data cut for analysis (March 1, 2020), only 1 patient from study remained alive and all patients were off study, including 15 patients (75%) due to progressive disease (6 clinical PD and 9 PD by RECIST) and 5 withdrew consent from study (3 patients due to toxicity and 2 patients decided to seek other treatment options).

## Discussion

Aberration in the function or expression of ERBB receptor tyrosine kinases contributes to tumorigenesis. EGFR or HER2 targeting agents are widely used and have shown substantial clinical efficacy. However, drug resistance often arises from aberrant or compensatory mechanism from downstream signaling proteins. Neratinib and trametinib have demonstrated clinical benefits when used as monotherapy or in combination with chemotherapy [26–31]. Furthermore, preclinical data have revealed synergistic effects of combination therapy with neratinib and trametinib including enhanced tumor inhibition [18].

Here, we are the first to examine the safety, toxicity and preliminary anti-tumor efficacy in patients with advanced solid tumors treated with neratinib and trametinib. Unfortunately, continuous daily dosing of neratinib and trametinib was poorly tolerated. Two patients experienced DLTs of diarrhea at the initial dose level resulting in dose de-escalation to dose level minus 1 which was neratinib at a dose of 160 mg once daily in combination with trametinib at a dose of 1 mg once daily on a 5 days on and 2 days off schedule. This cohort was expanded to include a minimum of six patients treated and evaluable for DLT to further evaluate safety and tolerability. In total, 8 patients were DLT evaluable, and dose level minus one was determined to be the MTD. The TRAEs observed in this study are consistent with previous studies and were mainly gastrointestinal toxicities such as diarrhea, nausea and vomiting [27, 32]. Monotherapy with neratinib at 180 mg daily displayed a similar toxicity profile and frequency with combination therapy on this study. Supp. Table 2 summarizes the TRAEs of all grades with neratinib or trametinib, and in combination. However, combination therapy revealed increased instances of grade 3 AEs including diarrhea, nausea, and vomiting as compared to either single agent neratinib or trametinib. This is likely due to overlapping toxicities causing additive side effects (Supp. Table 3). A previous study with dabrafenib and trametinib combination therapy has shown improvement in treatment tolerance when patients transitioned from continuous to intermittent dosing schedule [33]. Further exploration with intermittent dosing schedules of neratinib in combination with trametinib should be considered for future studies to help alleviate overlapping toxicity.

In our PK cohort of 8 patients treated at dose level minus 1 (160 mg neratinib daily with 1 mg trametinib 5 days on and 2 days off), trametinib pharmacokinetics, when co-administered with neratinib, appears to have a much lower oral clearance (1.55 L/h) leading to higher exposure (AUC_0-24_) compared to published single agent data results of 3.81 L/h [32, 34]. In fact, in our cohort, the mean trametinib exposure was 732 ng*h/mL ranging from 197 to 1054 ng*h/mL which was closer to the single agent dosing of trametinib at ≥ 2.5 mg [32]. Furthermore, at day 15, the accumulation ratios (AR) in our patient cohort are 2–3 times those reported by Infante et al. [32].

Studies of trametinib and neratinib have shown that each agent has different metabolism pathways. Neratinib is metabolized via cytochrome P450-3A4 (CYP3A4) pathway [35]. In our PK cohort, there was no observed changes in neratinib PKs compared to existing single agent PK data. However, neratinib is a known P-glycoprotein (P-gp) inhibitor and was shown to increase digoxin (a P-gp substrate) plasma concentrations (Cmax) by 54% and the AUC by 32% [36, 37]. Prior human study has shown that trametinib undergoes a non-cytochrome-mediated metabolism, involving deacetylation via hydrolytic enzymes alone or in combination with glucuronidation [38]. Trametinib is also a substrate of drug transporters, P-gp and breast cancer resistant protein (Bcrp) suggesting a P-gp and/or Bcrp substrate or inhibitor can modulate trametinib clearance and exposure [39]. Trametinib may be a low-level CYP3A4 inducer and, therefore, could lead to higher clearance of agents that are mainly metabolized by this same isoenzyme [38]. Fortunately, recent data revealed concomitant administration of trametinib and oral contraceptives (OC), well-known to be metabolized by CYP3A isoenzymes, showed no significant differences in the PK disposition of OC when compared to OC alone, and without any changes in clinical efficacy of OC [40].

We believe the observed drug–drug interaction from our PK analysis is due to combination therapy with neratinib and trametinib, with neratinib inhibiting the clearance of trametinib via the P-gp efflux pathway leading to a very high exposure of trametinib. There may be other factors such as saturation of deacetylation and glucuronidation enzyme pathways which may contribute to additional lowering of trametinib clearance resulting in increased total exposure. With trametinib as a substrate for P-gp, the use of P-gp strong inhibitors (e.g., ketoconazole, itraconazole, clarithromycin, erythromycin, ritonavir, verapamil) or P-gp inducers (e.g.,: phenytoin, rifampin, St. John’s wort, corticosteroids, efavirenz, nevirapine) should be avoided whenever possible when taking trametinib [41]. If clinically necessary, the use of known P-gp inhibitors or inducers when taking trametinib therapy, requires close monitoring and evaluation to avoid potential adverse events or decrease in clinical efficacy.

Limited anti-tumor efficacy was seen across all treated patients. The lack of meaningful response may be due to suboptimal dosing of both neratinib and trametinib. No partial responses were reported, and SD ≥ 4 months was seen in only 2 patients. Both patients were treated at dose level 1. No patients obtained SD ≥ 4 months or PR at dose level minus 1, the declared MTD.

Interestingly, both patients with SD ≥ 4 months harbored *EGFR* aberrations, including an ovarian cancer patient with *KRAS* G12D and *EGFR* G724S mutations and a salivary gland cancer patient with EGFR amplification by IHC. Zhao et al. showed that combination therapy with neratinib and trametinib induced reduction of ERK1/2 phosphorylation but failed to trigger robust anti-apoptotic activity. Zhao et al. also found that trametinib treatment resulted in downregulation of proteins involved in the MAPK and AKT pathways but increased total levels of EGFR [18]. This is in line with a prior study that correlated *EGFR* aberrations with favorable tyrosine kinase inhibitor treatment outcomes in lung cancer [42]. Taken together, these data may suggest that *EGFR* aberrations could be a response predictor for neratinib and trametinib treatment but the numbers in our study are too small to make any true assessment.

There are several limitations to this study. First, we were unable to dose escalate to meaningful doses of either agent in combination due to overlapping toxicity resulting in poor patient tolerance. Second, there was heterogeneity in the molecular aberrations allowable for enrollment onto study. Five of 11 (45.5%) patients enrolled on dose level minus 1 had only activating *KRAS* mutations with no co-occurring pan-*ERBB* aberrations. It is unclear if the lack of response at this dose level is due to suboptimal dosing versus the type of molecular aberration enrolled. Third, the sample size is small with patients being heavily pre-treated with a median of 3 lines of prior therapy and consisting of multiple solid tumor types making analysis challenging.

In conclusion, neratinib and trametinib combination therapy was not tolerable at dose level 1. MTD was declared as dose level minus 1 (neratinib 160 mg daily with trametinib 1 mg, 5 days on and 2 days off) and had limited clinical activity. This may be due to suboptimal drug dosing of trametinib and neratinib. The increased plasma exposure of trametinib may have contributed to the toxicity observed. Based on the results from our PK cohort, there was significant drug–drug interaction with an increase in trametinib plasma concentrations and exposure due to a decrease in clearance of trametinib. We hypothesize that this could be due to neratinib-induced inhibition of trametinib clearance via P-gp efflux mechanisms. Further work is needed in determining the best dose and/or schedule for treating patients with both agents in combination to improve tolerability. Additionally, further work needs to be done to determine the appropriate tumor types and molecular aberrations for enrollment.

## Supplementary Information

Below is the link to the electronic supplementary material.

Supplementary file1 (DOCX 14 KB)

Supplementary file2 (DOCX 14 KB)

Supplementary file3 (DOCX 14 KB)

## Data Availability

The dataset generated during and/or analyzed during the current study are available from the corresponding author on reasonable request.
