# Supplementary material for: A phase I trial of the pan-ERBB inhibitor neratinib combined with the MEK inhibitor trametinib in patients with advanced cancer with EGFR mutation/amplification, HER2 mutation/amplification, HER3/4 mutation or KRAS mutation
Source: Cancer Chemother Pharmacol. 2023 Jun 14;92(2):107–18. doi: 10.1007/s00280-023-04545-4 (PMC10326142; doi:10.1007/s00280-023-04545-4)
Supplement: Supplementary file 1 — Supplementary file1 (DOCX 14 KB) [file 280_2023_4545_MOESM1_ESM.docx]

**Supplementary Table1** Serious Adverse Events (SAEs)

| **SAE Term** | **Count** | **Attribution** |
| --- | --- | --- |
| Diarrhea | 6 | Related |
| Vomiting | 1 | Related |
| Nausea | 1 | Related |
| Anemia | 2 | Unrelated |
| Diarrhea | 1 | Unrelated |
| Dysphagia | 1 | Unrelated |
| Hepatobiliary disorders | 1 | Unrelated |
| Respiratory, thoracic and mediastinal disorders | 2 | Unrelated |
| Thromboembolic event | 1 | Unrelated |

Serious adverse events of all causality reported for patients on study. 8 events (diarrhea n=6; vomiting n=1; nausea n=1) were at least possibly related to the study drugs.
