# Supplementary material for: A phase I trial of the pan-ERBB inhibitor neratinib combined with the MEK inhibitor trametinib in patients with advanced cancer with EGFR mutation/amplification, HER2 mutation/amplification, HER3/4 mutation or KRAS mutation
Source: Cancer Chemother Pharmacol. 2023 Jun 14;92(2):107–18. doi: 10.1007/s00280-023-04545-4 (PMC10326142; doi:10.1007/s00280-023-04545-4)
Supplement: Supplementary file 2 — Supplementary file2 (DOCX 14 KB) [file 280_2023_4545_MOESM2_ESM.docx]

**Supplementary Table** **2** Common treatment-related adverse events of all grades (frequency >10%) with neratinib or trametinib, and in combination

|  | Neratinib (160mg) + Trametinib (1mg)  (N=20) | Neratinib (180mg)  (N=6)  (Wong et al)^27^ | Trametinib (2mg)  (N=70)  (Infante et al)^32^ |
| --- | --- | --- | --- |
| Diarrhea | 19 (95) | 6 (100) | 32 (46) |
| Nausea | 10 (50) | 3 (50) | 18 (26) |
| Vomiting | 6 (30) | 2 (33) | 9 (13) |
| Rash | 13 (65) | 1 (17) | 59 (84) |
| Fatigue | 4 (20) | 4 (67) | 21 (30) |
| Mucositis | 4 (20) | - | 3 (4) |
| Anorexia | 3 (15) | 1 (17) | - |
