# Supplementary material for: A phase I trial of the pan-ERBB inhibitor neratinib combined with the MEK inhibitor trametinib in patients with advanced cancer with EGFR mutation/amplification, HER2 mutation/amplification, HER3/4 mutation or KRAS mutation
Source: Cancer Chemother Pharmacol. 2023 Jun 14;92(2):107–18. doi: 10.1007/s00280-023-04545-4 (PMC10326142; doi:10.1007/s00280-023-04545-4)
Supplement: Supplementary file 3 — Supplementary file3 (DOCX 14 KB) [file 280_2023_4545_MOESM3_ESM.docx]

**Supplementary Table** **3** Grade > 3 treatment-related adverse events with neratinib or trametinib, and in combination

|  | Neratinib (160mg) + Trametinib (1mg)  (N=20) | Neratinib (180mg)  (N=6)  (Wong et al)^27^ | Trametinib (1mg)  (N=2)  (Infante et al)^32^ |
| --- | --- | --- | --- |
| Diarrhea | 5 (25) | 1 (17) | 0 |
| Nausea | 1 (5) | 0 | 0 |
| Vomiting | 2 (10) | 0 | 0 |
| Rash | 0 | 0 | 0 |
| Fatigue | 1 (5) | 1 (17) | 0 |
